# Supplementary material for: Cross-linking peptide and repurposed drugs inhibit both entry pathways of SARS-CoV-2
Source: Nat Commun. 2021 Mar 9;12:1517. doi: 10.1038/s41467-021-21825-w (PMC7943568; doi:10.1038/s41467-021-21825-w)
Supplement: Supplementary file 1 — Supplementary Information [file 41467_2021_21825_MOESM1_ESM.pdf]

Supplementary Information

Cross-linking peptide and repurposed drugs inhibit both entry pathways of SARS-CoV-2

Hanjun Zhao, Kelvin K. W. To, Hoiyan Lam, Xinxin Zhou, Jasper Fuk-Woo Chan, Peng Zheng, Andrew C. Y. Lee, Jianpiao Cai, Wan-Mui Chan, Jonathan Daniel Ip, Chris Chung-Sing Chan, Man Lung Yeung, Anna Jinxia Zhang, Allen Wing Ho Chu, Shibo Jiang, Kwok-Yung Yuen

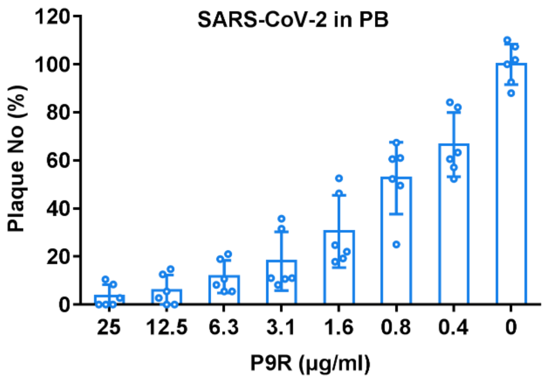

Supplementary Fig. 1. Antiviral activity of P9R in PB buffer. SARS-CoV-2 was pretreated by the indicated concentrations of P9R in 30 mM phosphate buffer (PB). After 45 min incubation, plaque reduction assay was used to measure the antiviral activity of P9R. Plaque number of virus treated by P9R was normalized to the plaque number of untreated virus. Data are presented as mean ±SD of five independent experiments. Source data are provided as a Source Data file.

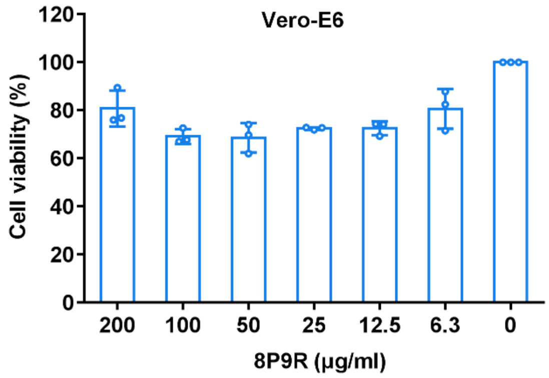

Supplementary Fig. 2. Cytotoxicity of 8P9R in Vero-E6. Vero-E6 cells were cultured in the presence of indicated concentrations of 8P9R in DMEM with 1% FBS medium. After 24h culture, MTT assay was used to measure the cell viability. Data are presented as mean ±SD from three independent experiments. Source data are provided as a Source Data file.

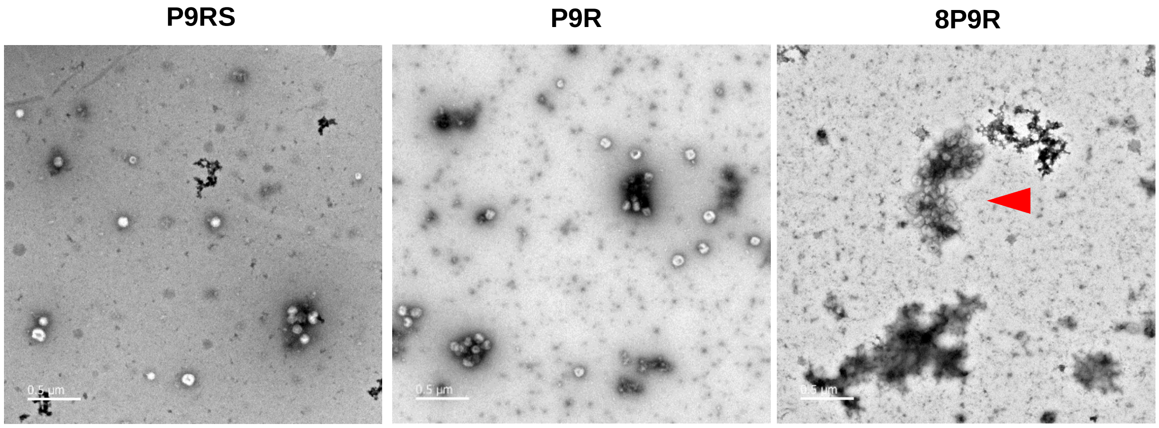

Supplementary Fig. 3. Cross-linking of SARS-CoV-2 by 8P9R. SARS-CoV-2 was treated by P9RS, P9R, or 8P9R (50 μg ml^{-1}) for 1h at room temperature. The treated virus was negatively stained for TEM assay. Red triangle indicated the big cluster of cross-linked SARS-CoV-2. Scale bar = 0.5 μm. Experiments were repeated twice. Source data are provided as a Source Data file.

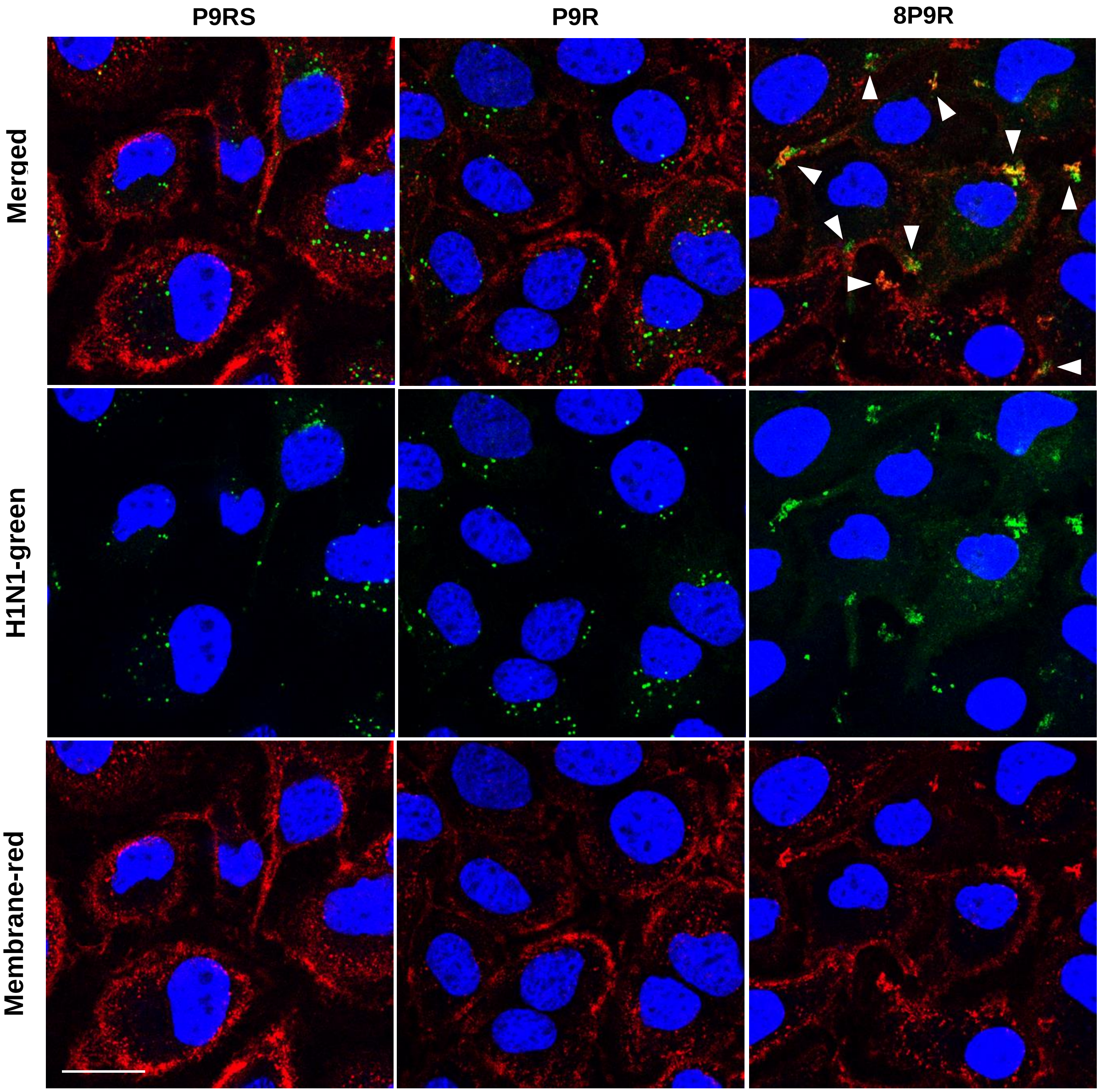

Supplementary Fig. 4. H1N1 virus was pre-labelled by green fluorescence dye and then treated by peptide P9RS, P9R or 8P9R (25 μg ml^{-1}) at room temperature for 45 min. Treated virus infected MDCK cells for 1h at 37°C and then cells were fixed and stained by cell membrane dye (red) and nuclear dye (blue). White triangles indicated the cross-linked viruses located at cell membrane. Scale bar = 20 μm. Experiments were repeated twice. Source data are provided as a Source Data file.

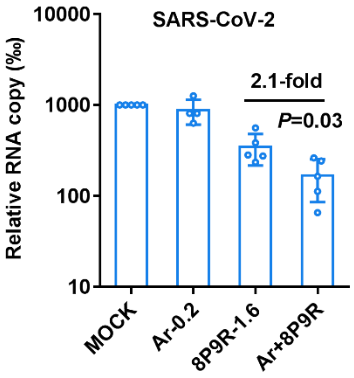

Supplementary Fig. 5. The enhanced antiviral activity of arbidol by 8P9R. SARS-CoV-2 was cultured in the presence of indicated arbidol (Ar-0.2, 0.2 μg ml^{-1}), 8P9R-1.6 (1.6 μg ml^{-1}) or the combination of Ar+8P9R. Viral titers in supernatants were measured at 24h post infection by RT-qPCR. Data are presented as mean ±SD from four independent experiments. *P* value was calculated by two-tailed student *t* test. Source data are provided as a Source Data file.

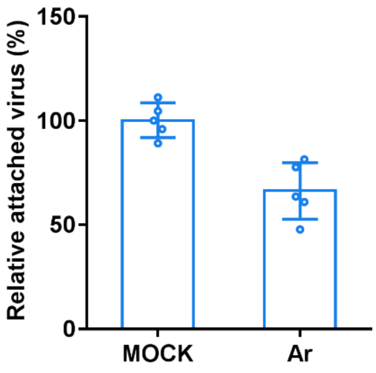

Supplementary Fig. 6. Arbidol could only reduce 30% of viral attachment of SARS-CoV-2 in Vero-E6 cells. SARS-CoV-2 was pretreated by arbidol (Ar, 25 μg ml^{-1}) or 0.1% DMSO (Mock) and then was added to Vero-E6 cells at 4℃for attachment. One hour later, the unattached virus was washed away. The attached virus was measured by RT-qPCR. Relative attached virus (%) was normalized to mock. Data are presented as mean ±SD from three independent experiments. Source data are provided as a Source Data file.

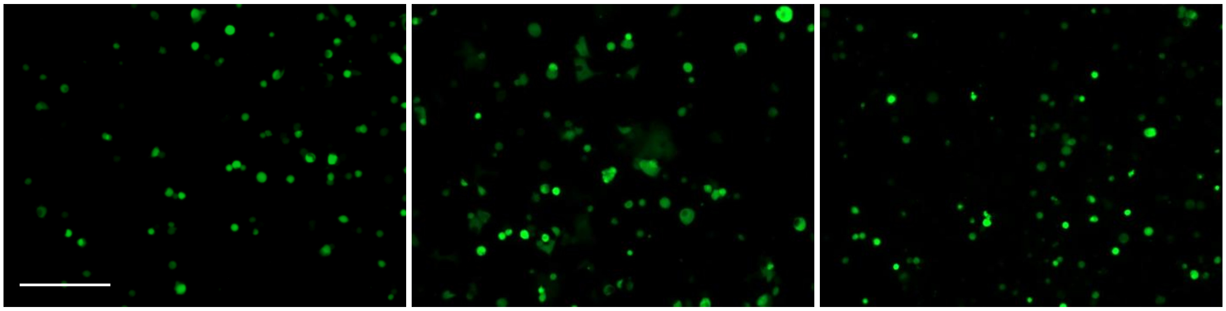

Supplementary Fig. 7. Arbidol could inhibit spike-ACE2 mediated cell membrane fusion in Huh7 cells. The 293T cells expressing spike+GFP were co-cultured with Huh7 cells at the presence of arbidol (50 μg ml^{-1}) or 0.2% DMSO (Mock). The 293T cells without spike were co-cultured with Huh7 cells was as the negative control of cell-cell fusion. Arbidol could block the cell fusion. The fused cell sizes were >2-fold bigger than the normal non-fused cells. Scale bar = 100 μm. The representative pictures were taken at 8 h after co-culture. Experiments were repeated twice. Source data are provided as a Source Data file.

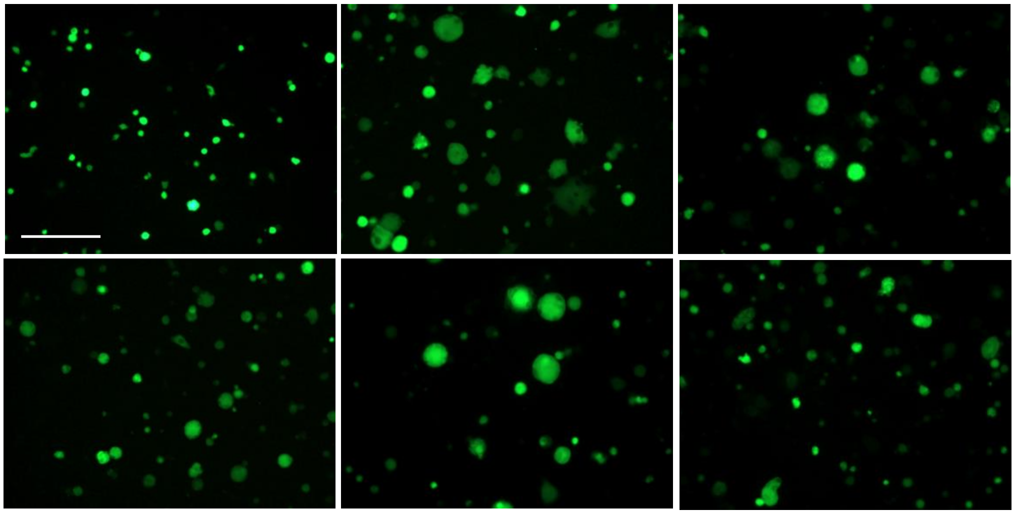

Supplementary Fig. 8. Combination of chloroquine and arbidol could more efficiently inhibit spike-ACE2 mediated cell-cell fusion. The 293T cells expressed ACE2 and spike-GFP were co-cultured in the presence of arbidol (25 μg ml^{-1}, Ar-25), chloroquine (100 μg ml^{-1}, Chl-100), chloroquine (25 μg ml^{-1}, Chl-25), or combination of Chl-25+Ar-25. 293T-GFP without spike (-Spike) served as the negative control of cell-cell fusion. Chl-25 and Ar-25 could not block the cell fusion, of which the fused cell sizes were >2-fold bigger than the normal non-fused cells. Combination of Chl-25 and Ar-25 could block the cell fusion. The representative pictures were taken at 8 h after co-culture. Scale bar = 100 μm. Experiments were repeated three times. Source data are provided as a Source Data file.

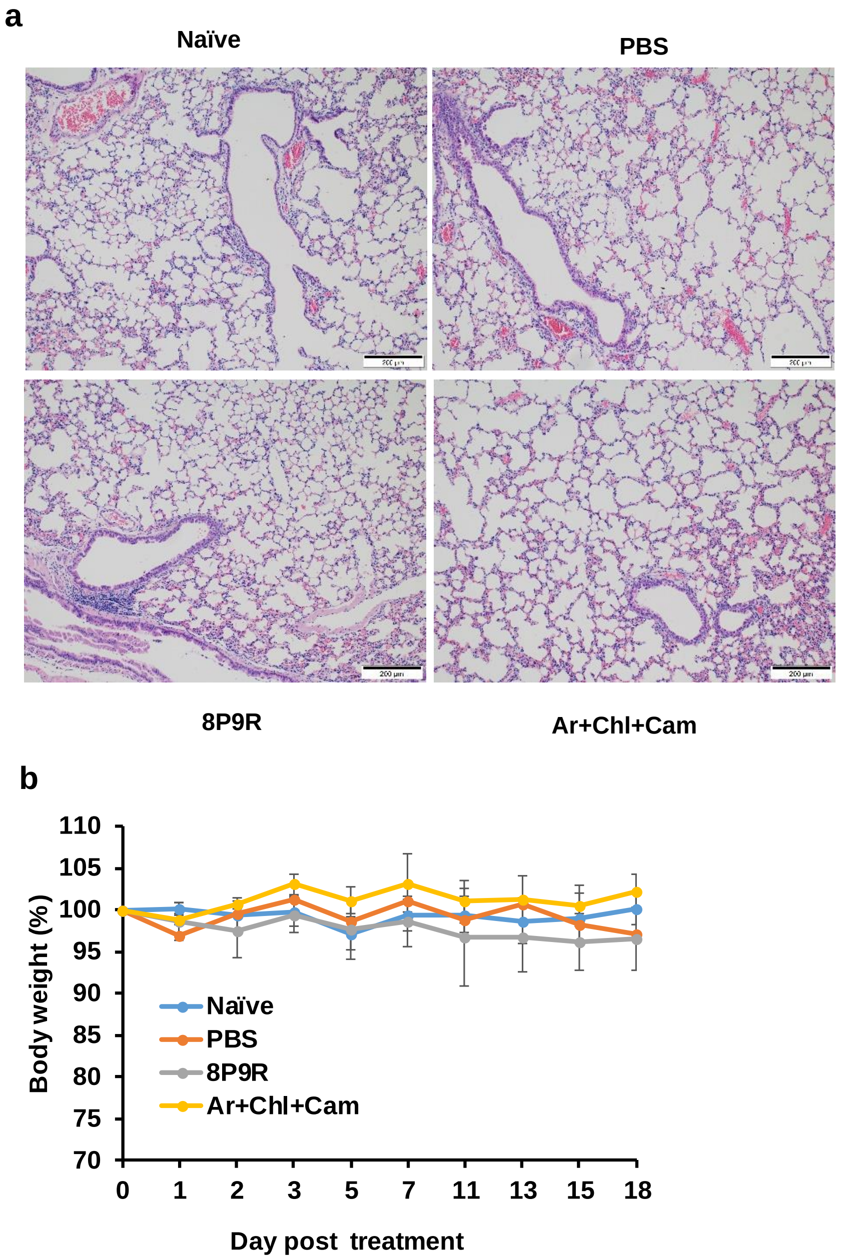

Supplementary Fig. 9. H&E staining and body weight of mice treated by drugs. Mice were intranasally inoculated with 8P9R (0.5mg/kg), camostat (0.3mg/kg), or orally inoculated with arbidol (30 mg/kg) and chloroquine (40 mg/kg). Two more doses were given to mice in the following day. (**a**) Lung tissues were harvested at day 2 for H&E staining from two independent experiments. (**b**) The body-weight changes of drug-treated mice were monitored for 18 days. Data are presented as mean ±SD from three mice. Source data are provided as a Source Data file.

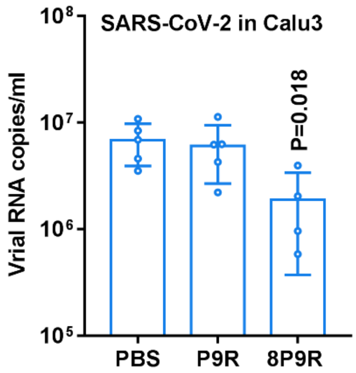

Supplementary Fig. 10. 8P9R could significantly inhibit SARS-CoV-2 replication in Calu-3 cells at post infection time. SARS-CoV-2 (0.1 MOI) infected Calu-3 cells. At 6 h post infection, peptides (50 µg ml^{-1}) were added to viral culture media. The viral RNA copies in supernatants were measured by RT-qPCR at 30 h post infection. *P* value was calculated by two-tailed student *t* test. Data are presented as mean ±SD of five independent biological samples. Source data are provided as a Source Data file.

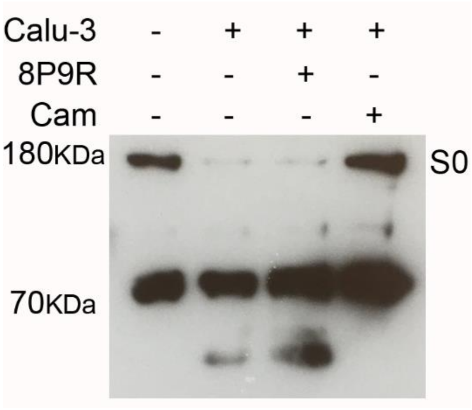

Supplementary Fig. 11. Camostat but not 8P9R could inhibit TMPRSS2 to cleave S protein. Calu-3 cells were pre-treated by camostat (Cam, 50 μg ml^{-1}), 8P9R (50 μg ml^{-1}) or PBS for 1h and then S protein (1 μg) of SARS-CoV-2 was added to treated cells for 1h to let TMPRSS2 to cleave the S protein. S protein without Calu-3 cell and drugs was served as the negative control. The samples were processed for western blot assay with anti-S2 antibody. Data indicated that 8P9R did not protect S protein cleavage in Calu-3 cells. Experiments were repeated twice. Source data are provided as a Source Data file.

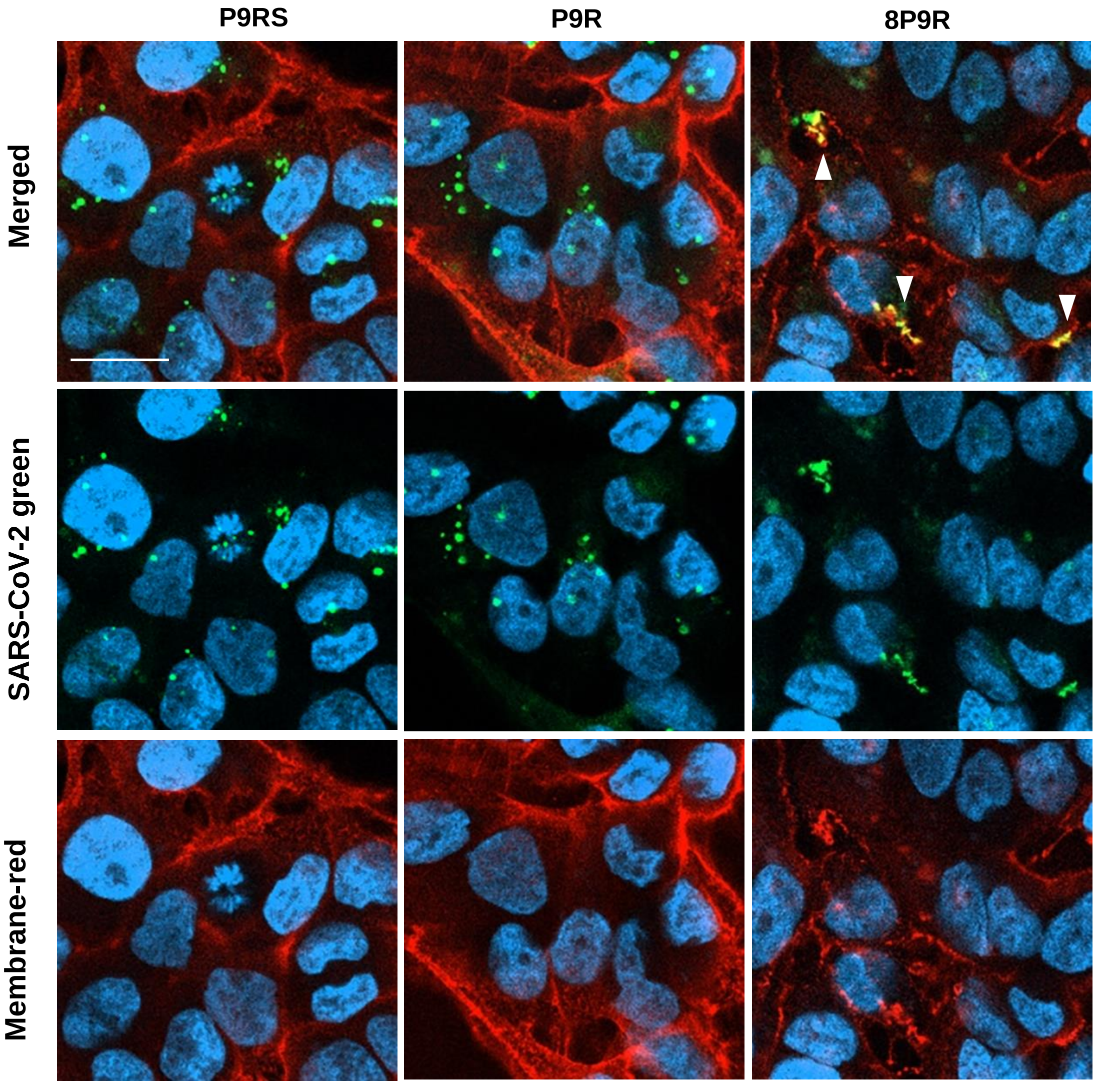

Supplementary Fig. 12. 8P9R could clustered SARS-CoV-2 in Calu-3 cells. SARS-CoV-2 virus was pre-labelled by green fluorescence dye and then treated by peptide P9RS, P9R or 8P9R (25 μg ml^{-1}) at room temperature for 45 min. Treated virus infected Calu-3 cells for 1h at 37°C and then cells were fixed and stained by cell membrane dye (red) and nuclear dye (blue). White triangles indicated the cross-linked viruses located at cell membrane. Scale bar = 20 μm. Represent images were taken by confocal microscope with 400× magnification. Source data are provided as a Source Data file.

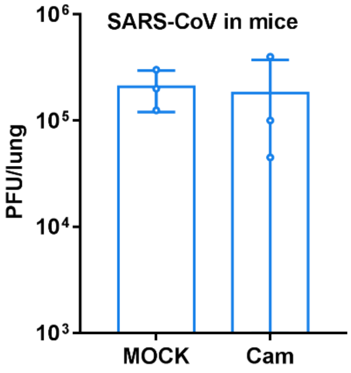

Supplementary Fig. 13. Camostat did not inhibit SARS-CoV replication in mice. Mice were intranasally inoculated with SARS-CoV (2×10^{3} PFU). Camostat (Cam: 15 mg kg^{-1}, n=3) or DMSO in PBS (Mock, n=3) was orally inoculated to mice at 8 h post infection. Two more doses were given to mice in the following one day. Lung tissues were collected at 2-day post infection. Viral loads in lungs were measure by plaque assay. Data are presented as mean ±SD of three mice. Source data are provided as a Source Data file.

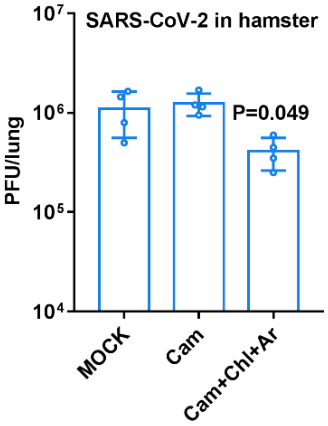

Supplementary Fig. 14. Drug combination of camostat, chloroquine and arbidol could inhibit SARS-CoV-2 replication in hamsters. Hamsters were intranasally inoculated with SARS-CoV-2 (5×10^{3} PFU). Camostat (Cam: 15 mg kg^{-1}, n=3), camostat+chloroquine+arbidol (Cam+Chl+Ar:15 mg kg^{-1} +40 mg kg^{-1} +30 mg kg^{-1}) or DMSO in PBS (Mock, n=4) was orally inoculated to mice at 8 h post infection. Two more doses were given to mice in the following one day. Lung tissues were collected at 2-day post infection. Viral loads in lungs were measure by plaque assay. Data are presented as mean ±SD of four hamsters. *P* value was calculated by two-tailed student *t* test when compared with Mock. Source data are provided as a Source Data file.

Supplementary Table 1. Primers of RT-qPCR.

Gene	Primer	Oligonucleotide sequence (5' to3')
SARS-CoV-2	S-F	CCTACTAAATTAAATGATCTCTGCTTTACT
	S-R	CAAGCTATAACGCAGCCTGTA
SARS-CoV	NP-F	ACCAGAATGGAGGACGCAAT
	NP-R	GCTGTGAACCAAGACGCAGTATTAT
